# Production of butyric acid from acid hydrolysate of corn husk in fermentation by *Clostridium tyrobutyricum*: kinetics and process economic analysis

**DOI:** 10.1186/s13068-018-1165-1

**Published:** 2018-06-15

**Authors:** Zhiping Xiao, Chu Cheng, Teng Bao, Lujie Liu, Bin Wang, Wenjing Tao, Xun Pei, Shang-Tian Yang, Minqi Wang

**Affiliations:** 1 College of Animal Science, Zhejiang University, No. 866 Yuhangtang Road, Hangzhou, 310058 People’s Republic of China; 2 Department of Chemical and Biomolecular Engineering, The Ohio State University, 151 West Woodruff Avenue, Columbus, OH 43210 USA

**Keywords:** Acid hydrolysis, Butyric acid, *Clostridium tyrobutyricum*, Corn husk, Fermentation, Fibrous-bed bioreactor

## Abstract

**Background:**

Butyric acid is an important chemical currently produced from petrochemical feedstocks. Its production from renewable, low-cost biomass in fermentation has attracted large attention in recent years. In this study, the feasibility of corn husk, an abundant agricultural residue, for butyric acid production by using *Clostridium tyrobutyricum* immobilized in a fibrous bed bioreactor (FBB) was evaluated.

**Results:**

Hydrolysis of corn husk (10% solid loading) with 0.4 M H_2_SO_4_ at 110 °C for 6 h resulted in a hydrolysate containing ~ 50 g/L total reducing sugars (glucose:xylose = 1.3:1.0). The hydrolysate was used for butyric acid fermentation by *C. tyrobutyricum* in a FBB, which gave 42.6 and 53.0% higher butyric acid production from glucose and xylose, respectively, compared to free-cell fermentations. Fermentation with glucose and xylose mixture (1:1) produced 50.37 ± 0.04 g L^−1^ butyric acid with a yield of 0.38 ± 0.02 g g^−1^ and productivity of 0.34 ± 0.03 g L^−1^ h^−1^. Batch fermentation with corn husk hydrolysate produced 21.80 g L^−1^ butyric acid with a yield of 0.39 g g^−1^, comparable to those from glucose. Repeated-batch fermentations consistently produced 20.75 ± 0.65 g L^−1^ butyric acid with an average yield of 0.39 ± 0.02 g g^−1^ in three consecutive batches. An extractive fermentation process can be used to produce, separate, and concentrate butyric acid to > 30% (w/v) sodium butyrate at an economically attractive cost for application as an animal feed supplement.

**Conclusion:**

A high concentration of total reducing sugars at ~ 50% (w/w) yield was obtained from corn husk after acid hydrolysis. Stable butyric acid production from corn husk hydrolysate was achieved in repeated-batch fermentation with *C. tyrobutyricum* immobilized in a FBB, demonstrating that corn husk can be used as an economical substrate for butyric acid production.

## Background

Butyric acid is a four-carbon volatile fatty acid with wide applications in chemical, food, animal feed, and pharmaceutical industries [1, 2] with an annual market of more than 80,000 metric tons globally [3]. Butyric acid is the primary energy source in intestinal metabolism, and have therapeutic effects on tumor growth, immune system, and gastrointestinal diseases [4–6]. Butyric acid can also be used to produce esters such as ethyl butyrate and butyl butyrate for use as solvents and fragrance in perfume [7]. Current butyric acid production from petroleum feedstocks is unsustainable and causes environmental concerns [1]. Using renewable biomass as the feedstock to produce butyric acid via fermentation can provide an attractive alternative and alleviate concerns of future scarcity and environmental impacts of fossil fuels.

Naturally, many anaerobic microorganisms can produce butyric acid from sugars and other carbon sources [1]. Particularly, several *Clostridium* species including *C. butyricum, C. thermobutyricum*, and *C. tyrobutyricum* can produce butyric acid as the main metabolic product and their potential for industrial production of butyric acid has been extensively studied [8–11]. Recent fermentation process studies for bio-production of butyric acid have focused on *C. tyrobutyricum* using various substrates, including glucose, xylose [12–14], sucrose [15], cane molasses [16], corn meal [17], Jerusalem artichoke [18], and brown algae [19]. Since carbon source accounts for a large proportion of raw material costs, second-generation biorefineries focus on using abundant, cheap, renewable lignocellulosic biomass to produce biofuels and bio-based chemicals, including butyric acid [8, 20, 21]. The feasibility of using agricultural residues, including wheat straw [22], corn fiber [23], oilseed rape straw [24], and sugarcane bagasse [25], for butyric acid production have been studied and reported.

However, conventional butyric acid fermentation suffers from a low product yield because of simultaneous production of acetic acid as a by-product and low productivity due to inhibition by butyric acid [26, 27]. These problems were partially solved in fermentation with cells immobilized in a fibrous bed bioreactor (FBB) [28], which not only significantly increased cell density and productivity, but also greatly improved product yield and titer with reduced acetic acid production [29–31].

In this study, we evaluated the feasibility of using corn husk, an agricultural residue obtained from the corn field, as the feedstock for butyric acid production. Every year huge amounts of corn husk are left as waste after harvesting corn. Currently, corn husk has little use and is usually burnt in the field, causing significant air pollution [32]. However, this environmentally problematic waste can be used as a low-cost feedstock after acid hydrolysis to reducing sugars, mainly glucose and xylose [33], for butyric acid production in fermentation by *C. tyrobutyricum*, as demonstrated in the present study. Based on the fermentation kinetics data, a cost analysis was also performed for producing sodium butyrate from corn husk in an extractive fermentation process.

## Results

### Preparation of corn husk hydrolysate

Acid or alkali pretreatment at high temperatures is widely used to facilitate the hydrolysis of lignocellulosic biomass [34, 35]. To identify the optimum conditions for acid hydrolysis of corn husk, four key process factors (temperature, acid concentration, treatment time, and corn husk to sulfuric acid ratio) were evaluated for their effects on the hydrolysis of corn husk to total reducing sugars. Each factor was studied at 4 levels in an orthogonal design for a total of 16 experiments (4 factors × 4 levels), and the concentrations of total reducing sugars obtained were then used to calculate *k* and *R* values, which indicate the importance of factors studied. A larger *R* value indicates a greater effect of the factor on the process. As can be seen in Table 1, increasing the hydrolysis temperature had the greatest effect on increasing the resulting total reducing sugars concentration, followed by the acid concentration and hydrolysis time, while increasing the corn husk to sulfuric acid ratio showed a negligible effect on increasing the total reducing sugars concentration. Considering the hydrolysis efficiency and sugar yield from corn husk, the optimum condition was determined as follows: acid concentration 0.4 M, temperature 120 °C, hydrolyzing time 6 h, and corn husk to sulfuric acid ratio 1:10 (w:v). Under this condition, a high total reducing sugars concentration of 50.67 g L^−1^ was obtained in the corn husk hydrolysate.

**Table 1:** The orthogonal design and analysis of acid hydrolysis of corn husk at four different levels of acid concentration, corn husk to sulfuric acid ratio, time, and temperature

No.	Factor				Total reducing sugars (g L^−1^)
	Acid concentration (M)	Corn husk to sulfuric acid (w:v)	Time (h)	Temperature (°C)	
1	0.2	1:10	2	90	4.83
2	0.2	1:15	4	100	12.36
3	0.2	1:20	6	110	16.48
4	0.2	1:25	8	120	32.81
5	0.3	1:10	4	110	21.59
6	0.3	1:15	2	120	36.82
7	0.3	1:20	8	90	11.93
8	0.3	1:25	6	100	13.92
9	0.4	1:10	6	120	50.67
10	0.4	1:15	8	110	28.55
11	0.4	1:20	2	100	12.39
13	0.5	1:10	8	100	18.86
15	0.5	1:20	4	120	40.0
16	0.5	1:25	2	110	25.43
Value					
*k*1	16.62	23.99	19.87	11.43	
*k*2	21.07	23.87	21.29	14.38	
*k*3	25.71	20.20	24.70	23.01	
*k*4	25.51	20.85	23.04	40.08	
*R*	9.09	3.79	4.83	28.65	

*k*1–*k*4 values are the mean values of total reducing sugars for each factor at levels 1–4, respectively; *R* is the range or difference between the maximum and minimum *k* values for each factor. A larger *R* value indicates a greater effect of the factor on the process

**Fig. 1:**
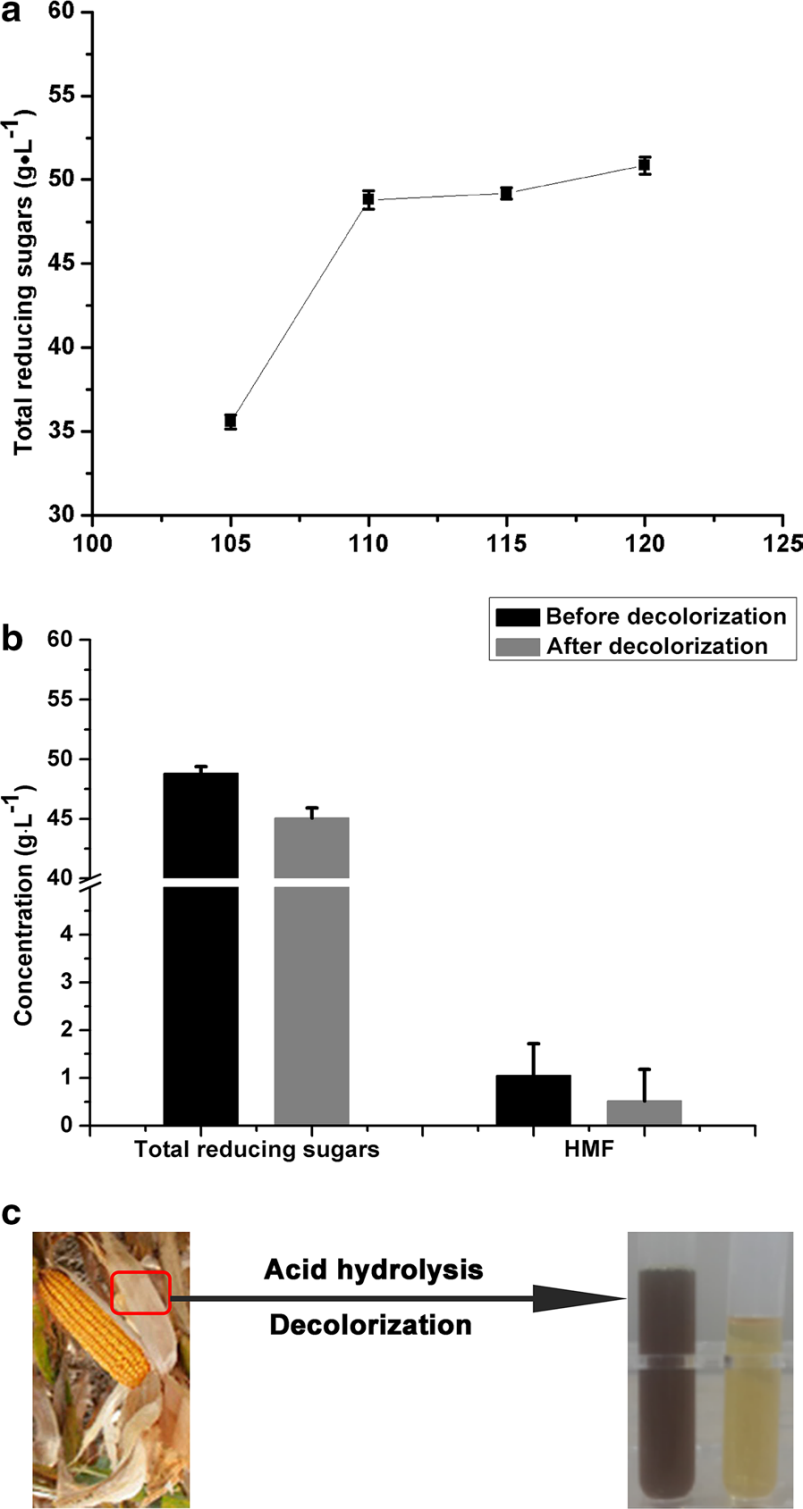
Preparation of corn husk hydrolysate. **a** Total reducing sugars released from corn husk treated with 0.4 M H_2_SO_4_ at various temperatures for 6 h, with 10% (w/v) solid loading; **b** concentrations of total reducing sugars and HMF before and after detoxification of corn husk hydrolysate. **c** Corn husk hydrolysate with color changed from dark brown to pale yellow after detoxification. Data are represented as mean ± standard deviation with *n* = 3

Acid hydrolysis of corn husk was further studied with 0.4 M H_2_SO_4_ containing 10% (w/v) corn husk at different temperatures (105, 110, 115, and 120 °C) for the treatment time of 6 h. As shown in Fig. 1a, total reducing sugars increased with increasing the temperature and reached 48.81 ± 0.55 g L^−1^ at 110 °C. The results of HPLC showed that the major components of the corn husk hydrolysate were glucose and xylose with the ratio of ~ 1.3:1.0. In addition, 1.046 ± 0.67 g L^−1^ 5-hydroxymethylfurfural (HMF), the product derived from glucose dehydration in the acid treatment, was also present in the hydrolysate. Further increasing the temperature to 115 and 120 °C had little effect on increasing total reducing sugars. Glucose and xylose can be degraded under high temperature conditions, which not only reduced sugar yield but also generated toxic products inhibiting the fermentation [25]. Therefore, acid pretreatment of corn husk with 10% (w/v) solid loading in 0.4 M H_2_SO_4_ at 110 °C for 6 h was recommended and used in the subsequent experiments.

Macroporous activated carbon or resins can be used to decolorize and detoxify the hydrolysate by adsorption of HMF and other inhibitors such as phenolics derived from lignin degradation [36]. Therefore, detoxification was conducted after hydrolysis. After detoxification, the HMF content decreased 50.86% to 0.514 ± 0.66 g L^−1^, while the total reducing sugars decreased 7.68% to 45.06 ± 0.87 g L^−1^ (Fig. 1b). The color of the hydrolysate changed from dark brown to pale yellow after detoxification (Fig. 1c), indicating that some color compounds including phenolics were also removed in the detoxification process. The detoxified hydrolysate was thus used in fermentation studies.

### Butyric acid production from glucose and xylose

**Fig. 2:**
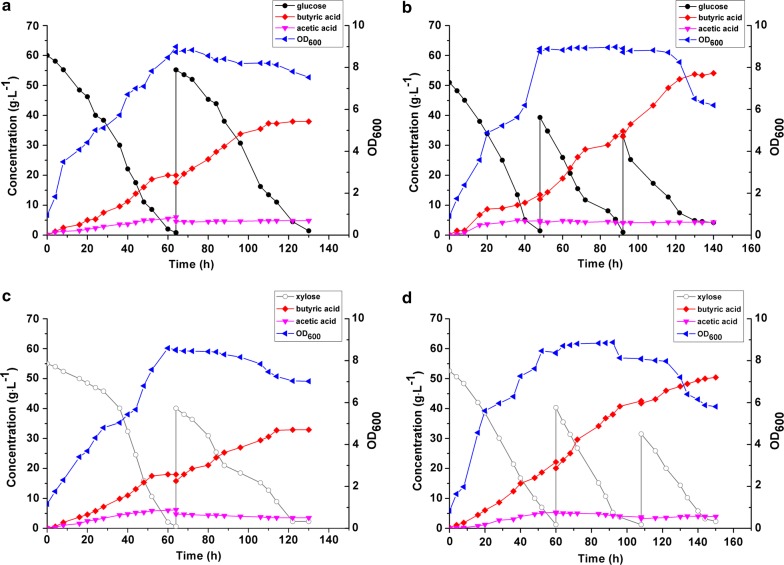
Butyric acid production from glucose and xylose, respectively, as carbon source in free-cell and FBB fermentations. The fermentation was operated at the fed-batch mode to reach the maximum butyric acid concentration. **a** Free-cell fermentation of glucose; **b** FBB fermentation of glucose; **c** free-cell fermentation of xylose; **d** FBB fermentation of xylose

To evaluate the feasibility of using corn husk hydrolysate for butyric acid production, glucose and xylose, the two main sugar components in the hydrolysate, as carbon sources were first studied in free-cell and immobilized-cell fermentations (Fig. 2). In general, butyric and acetic acids were simultaneously produced and reached 20.01 and 6.01 g L^−1^, respectively, from glucose (Fig. 2a) and 18.01 and 6.23 g L^−1^, respectively, from xylose (Fig. 2c) in 64 h in free-cell fermentations. After adding additional sugars, butyric acid production continued and reached the peak values of 37.95 ± 0.02 and 32.96 ± 0.05 g L^−1^, respectively, at 130 h, with the corresponding yield of 0.33 ± 0.03 and 0.32 ± 0.04 g g^−1^ from glucose and xylose, respectively. While butyric acid production continued with additional sugars added to reach a higher concentration level, acetic acid production and cell growth ceased due to inhibition by butyric acid [37, 38]. In anaerobic metabolism, more ATP can be produced in acetic acid biosynthesis than in butyric acid biosynthesis. Therefore, acetic acid production was better suited to meet the energy demand of rapid cell growth, but was inhibited by butyric acid and stopped when cell growth ceased [14]. The fermentation with cells immobilized in the FBB showed similar kinetics but reached a much higher butyric acid concentration of 54.12 ± 0.02 g L^−1^ (vs. 37.95 ± 0.02 g L^−1^) from glucose (Fig. 2b) and 50.43 ± 0.07 g L^−1^ (vs. 32.96 ± 0.05 g L^−1^) from xylose (Fig. 2d) as compared with free-cell fermentations.

Table 2 summarizes and compares the kinetics data in these fermentations. Compared to free-cell fermentation, butyric acid production from glucose and xylose in the FBB increased 42.6 and 53.0%, respectively, in the final titer, 36.4 and 28.1%, respectively, in butyric acid yield, and 34.5 and 36.0%, respectively, in the productivity because of increased tolerance to butyric acid inhibition [14, 28, 29]. The increased product yield was attributed to reduced cell growth and acetate production in fermentation with cells immobilized in the FBB.

**Table 2:** Comparison between free-cell and immobilized cell fermentations for butyric acid production from different carbon sources

Substrate	Glucose	Xylose			Mixture^a^	CHH
Fermentation mode	Free-cell	FBB	Free-cell	FBB	FBB	FBB
Total sugar consumed (g L^−1^)	113.80 ± 0.12	119.12 ± 0.25	103.82 ± 0.34	122.15 ± 0.13	133.95 ± 0.32	53.27 ± 0.21
Butyrate conc. (g L^−1^)	37.95 ± 0.02	54.12 ± 0.02	32.96 ± 0.05	50.43 ± 0.07	50.37 ± 0.04	20.75 ± 0.65
Butyrate yield (g g^−1^)	0.33 ± 0.03	0.45 ± 0.02	0.32 ± 0.04	0.41 ± 0.03	0.38 ± 0.02	0.39 ± 0.02
Butyrate productivity (g L^−1^ h^−1^)	0.29 ± 0.02	0.39 ± 0.01	0.25 ± 0.01	0.34 ± 0.03	0.34 ± 0.03	0.42 ± 0.06
Acetate conc. (g L^−1^)	4.83 ± 0.03	4.31 ± 0.04	3.50 ± 0.21	3.94 ± 0.04	4.08 ± 0.02	4.13 ± 0.22
Acetate yield (g g^−1^)	0.04 ± 0.02	0.04 ± 0.01	0.03 ± 0.01	0.03 ± 0.01	0.03 ± 0.02	0.08 ± 0.01
Butyrate/Acetate (g g^−1^)	7.86 ± 0.02	12.56 ± 0.04	9.41 ± 0.03	12.80 ± 0.07	12.30 ± 0.03	5.02 ± 0.24

Each value was the average of three replicate runs reported as mean ± standard deviation

*CHH* corn husk hydrolysate

^a^Mixture: 28.06 g L^−1^ glucose, 29.22 g L^−1^ xylose

**Fig. 3:**
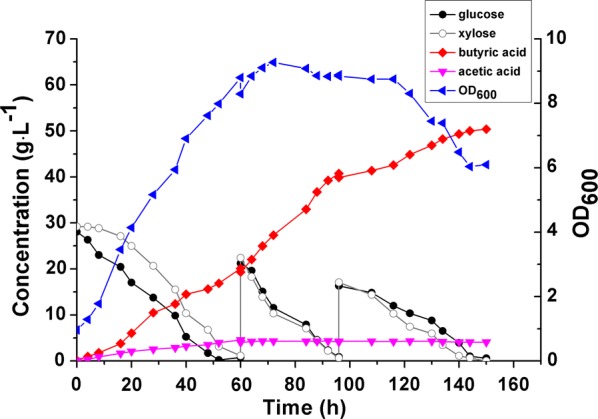
Fed-batch fermentation for butyric acid production from the mixture of glucose and xylose with the ratio of 1:1 (w:w) in the FBB

To investigate whether *C. tyrobutyricum* could consume glucose and xylose simultaneously, a mixture of these two sugars at the 1:1 ratio was used as carbon source in FBB fermentation. As shown in Fig. 3, initially xylose consumption by *C. tyrobutyricum* was repressed by glucose, but after 24 h both xylose and glucose were used simultaneously at almost equal rate in the fed-batch fermentation, producing 50.37 ± 0.04 g L^−1^ butyric acid (yield 0.38 ± 0.02 g g^−1^) from the sugar mixture.

### Butyric acid production from corn husk hydrolysate in FBB

**Fig. 4:**
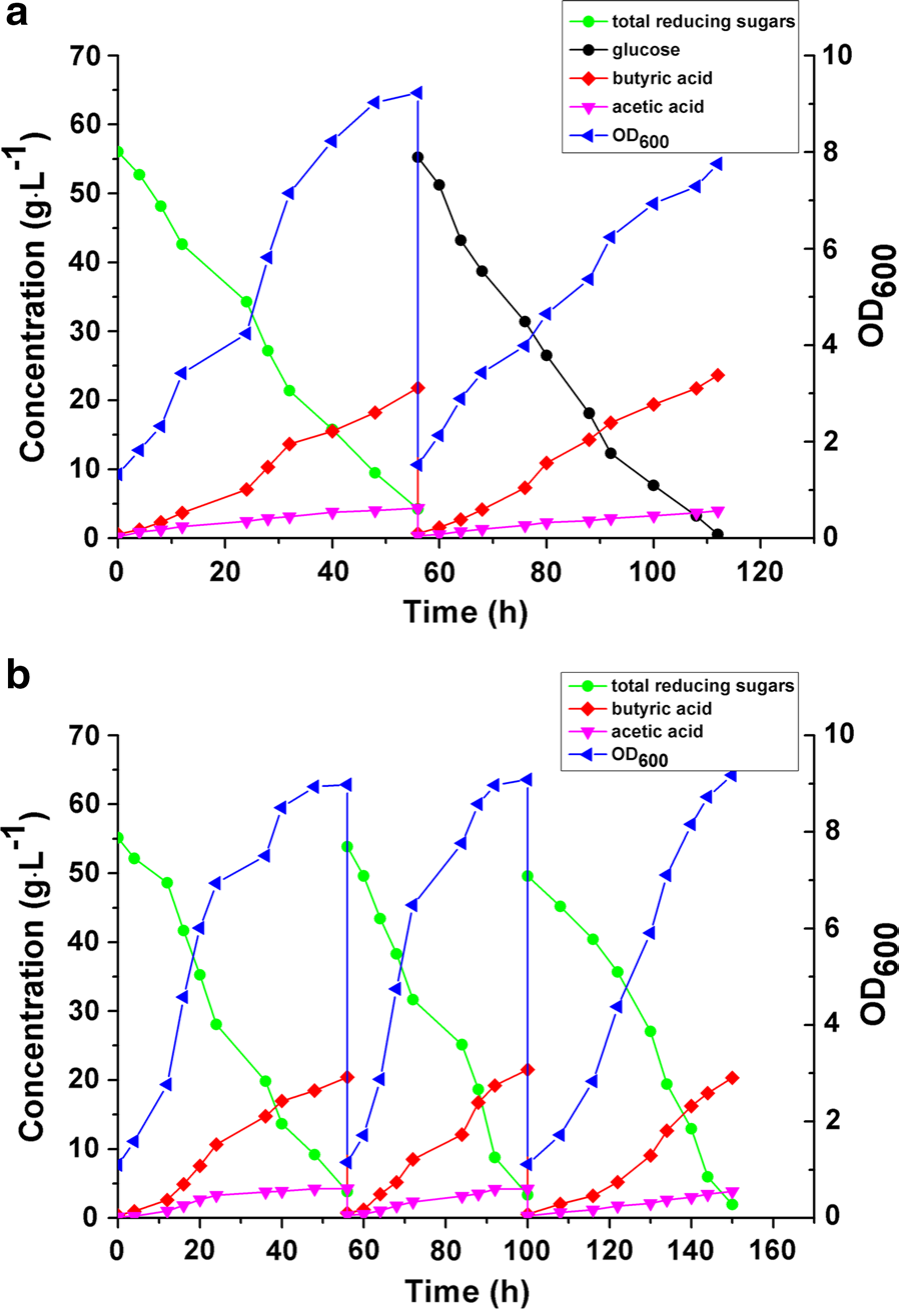
Repeated batch Fermentations for butyric acid production from corn husk hydrolysate by *C. tyrobutyricum* in the FBB. **a** Fermentation kinetics for butyric acid production from corn husk hydrolysate (first batch) and glucose (second batch). **b** Butyric acid production from corn husk hydrolysate in three consecutive batches

Fermentation of corn husk hydrolysate containing 31.80 g L^−1^ glucose and 24.24 g L^−1^ xylose was studied in the FBB. As shown in Fig. 4a, 21.80 g L^−1^ butyric acid and 4.33 g L^−1^ acetic acid were produced in 56 h, with a butyric acid yield of 0.39 g g^−1^ total reducing sugars consumed. For comparison, the FBB was then fed with a fresh medium containing glucose as the carbon source. In 56 h, 23.64 g L^−1^ butyric acid and 3.97 g L^−1^ acetic acid were produced. As can be seen in Fig. 4a, the fermentation kinetics was similar for corn husk hydrolysate and glucose, although the latter produced 8.4% more butyric acid with a 10.3% higher butyric acid yield (0.43 g g^−1^ vs. 0.39 g g^−1^). The slightly lower butyric acid production and yield from the hydrolysate could be attributed to the xylose, which is less energy efficient than glucose as discussed earlier. It should be noted that butyric acid production from the hydrolysate had comparable yield and productivity to those from the mixed sugars (glucose:xylose 1:1) (see Table 2), confirming that the detoxified corn husk hydrolysate was clean and good for butyric acid fermentation.

To further test the hydrolysate for possible long-term toxicity on *C. tyrobutyricum*, three consecutive batches were operated with the FBB. As shown in Fig. 4b, consistent and stable butyric acid production from the hydrolysate was observed for all three batches. Butyric acid production reached 20.44 g L^−1^ and acetic acid reached 4.28 g L^−1^ in 56 in the first batch. The fermentation time shortened to less than 50 h in the subsequent two batches because of the increased cell density in the FBB, which also resulted in increased butyric acid yield (from 0.37 g g^−1^ in the first batch to 0.41 g g^−1^ in the third batch) with a lower acetate production (3.87 g L^−1^ in the third batch). The average butyric acid yield from these three batches was 0.39 ± 0.02 g g^−1^ and productivity was 0.42 ± 0.06 g L^−1^ h^−1^.

### Process design and cost analysis for sodium butyrate production from corn husk

**Fig. 5:**
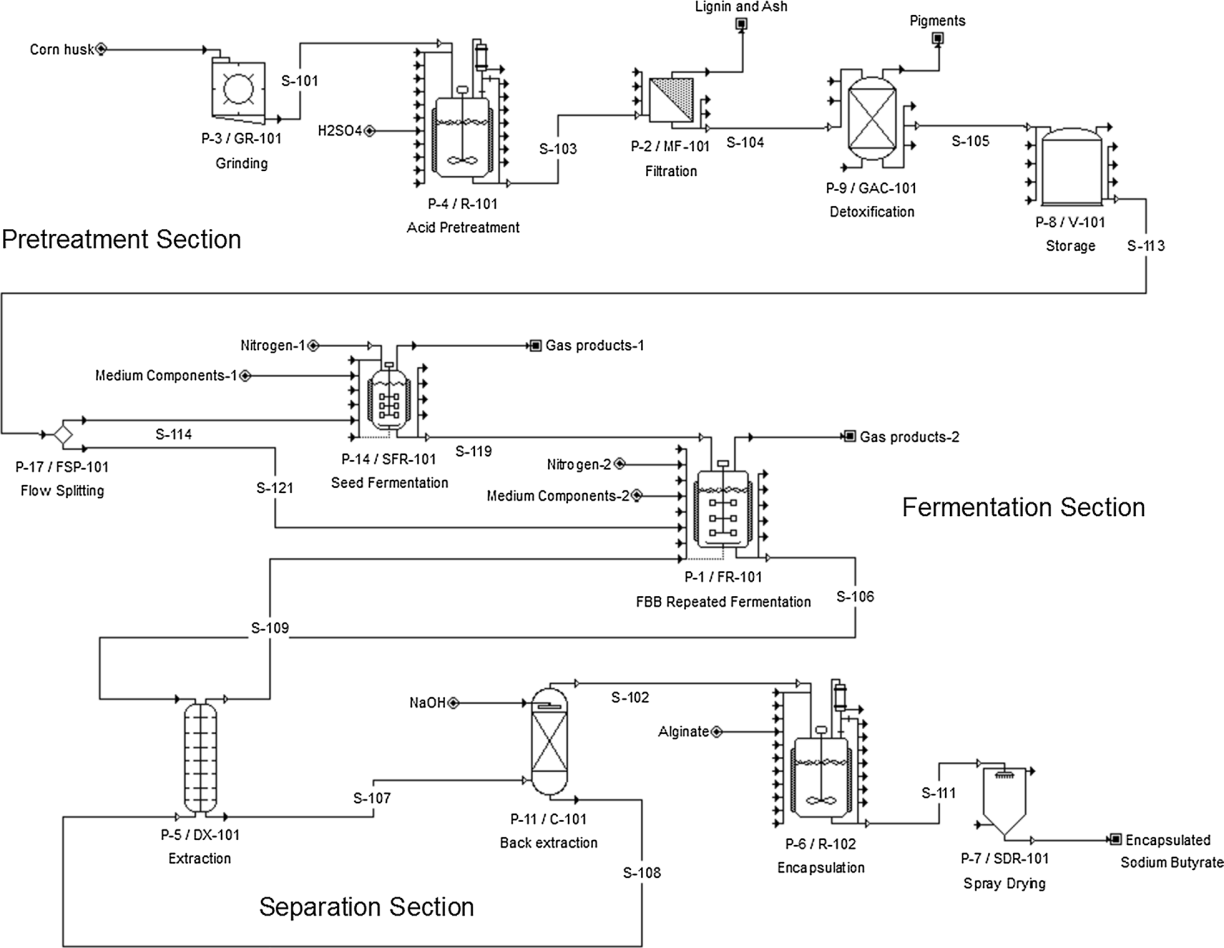
Process flowsheet for the production of encapsulated sodium butyrate from corn husk in an extractive fermentation process

The process for manufacturing sodium butyrate from corn husk includes pretreatments with dilute sulfuric acid for hydrolysis of corn husk polysaccharides (cellulose and hemicellulose) to fermentable sugars (mainly glucose and xylose) followed with detoxification with activated carbon adsorption to remove hydrolysate inhibitors, fermentation to convert sugars to butyric acid with *C. tyrobutyricum* in bioreactors (FBB), and downstream processing to separate butyric acid from the fermentation broth by solvent extraction and back extraction with NaOH to produce the final product. A previous study has shown that a highly concentrated sodium butyrate solution (> 300 g L^−1^) with a high purity (91% butyrate and 9% acetate) could be produced from glucose in an extractive fermentation process, which also gave higher reactor productivity (7.37 g L^−1^ h^−1^) and butyric acid yield (0.45 g g^−1^ glucose) than those from the conventional batch fermentation processes [37]. As butyric acid has a strong offensive odor, the sodium butyrate to be used as supplement in animal feed must be encapsulated (by mixing with gelatin and maltodextrin) and then spray-dried to pellets. Figure 5 shows the process flowsheet for manufacturing encapsulated sodium butyrate from corn husk.

**Fig. 6:**
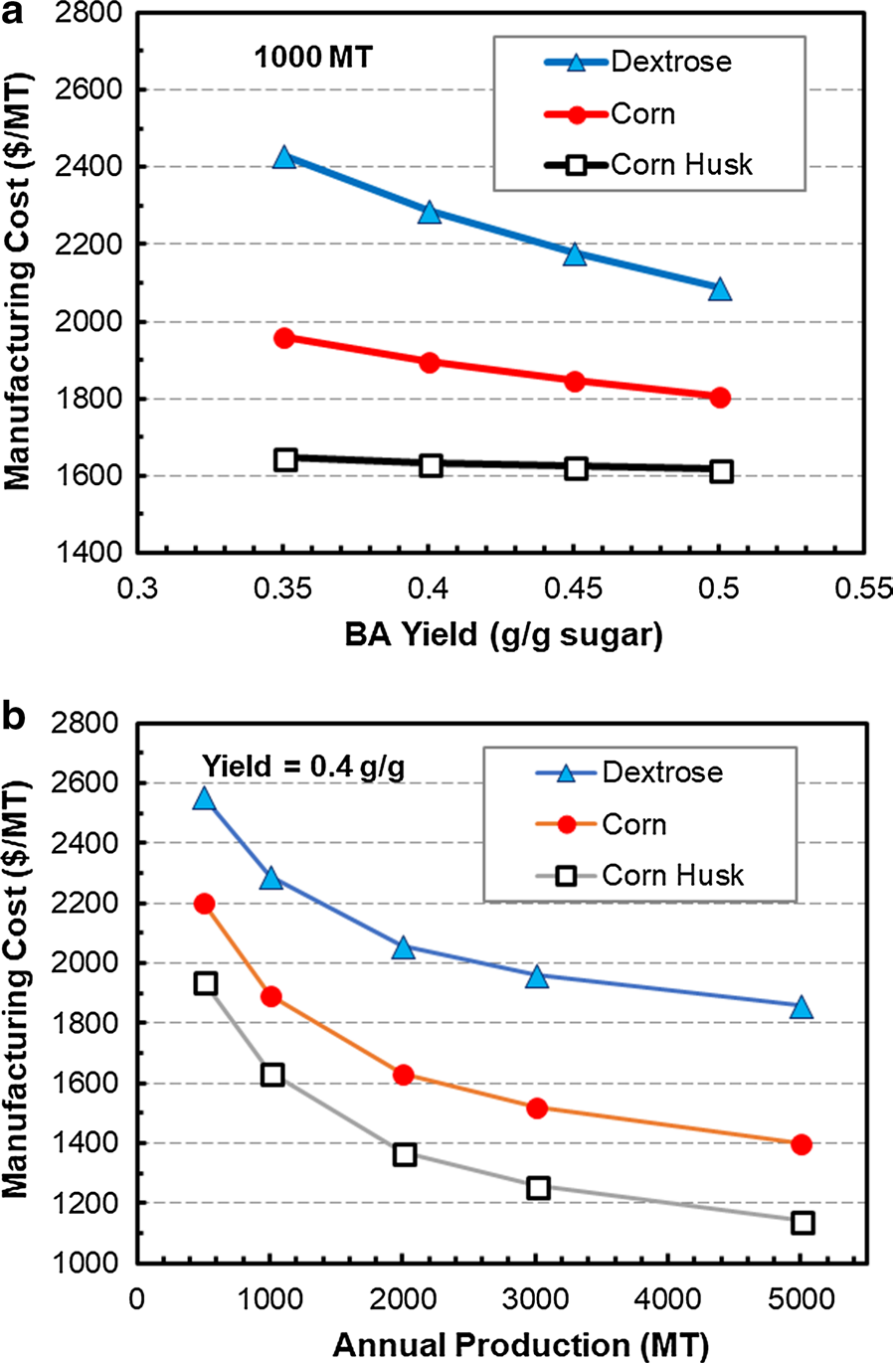
Effects of butyric acid yield and production scale on manufacturing costs of sodium butyrate (30% w/v) from dextrose, corn, and corn husk. **a** Effect of butyric acid yield on manufacturing cost. **b** Effect of production scale on manufacturing cost

**Fig. 7:**
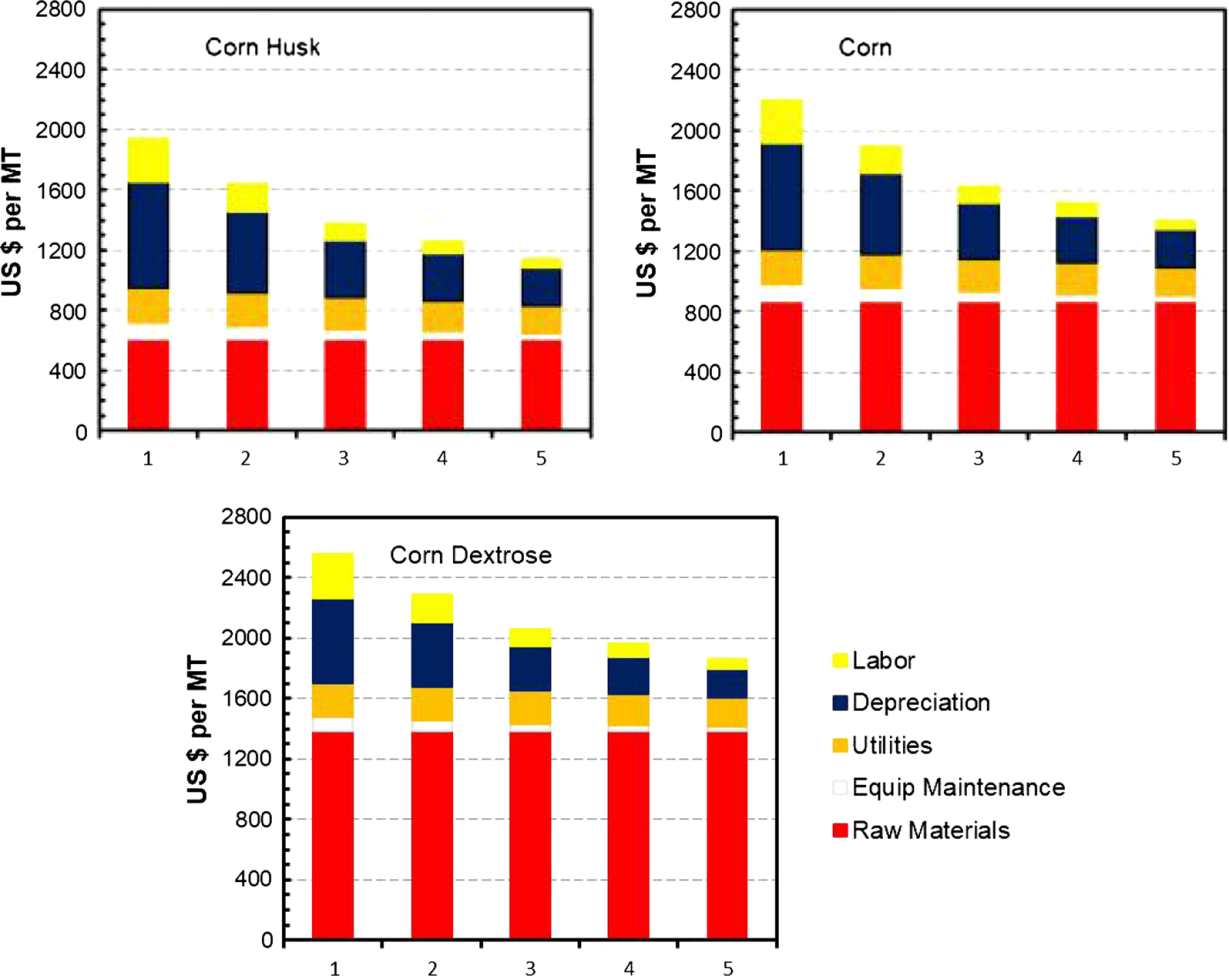
Analysis of manufacturing costs of sodium butyrate (30% w/v) in an extractive fermentation process using different substrates

The economic feasibility for producing sodium butyrate from corn husk for animal feed application is analyzed using SuperPro Designer. The current animal feed market for encapsulated sodium butyrate (~ 30% weight content) is about 15,000 MT annually in China alone. Figure 6 shows the costs for producing concentrated sodium butyrate (30% w/v) in the extractive fermentation process from corn husk, corn, and corn dextrose, respectively, as affected by the butyric acid yield (0.35–0.5 g g^−1^) from sugars in fermentation and production scale (500–5000 MT). In general, the manufacturing cost is sensitive to the butyric acid yield when dextrose and corn are used as the feedstock, but not much for corn husk (Fig. 6a). Corn husk as the least expensive feedstock accounts for only ~ 5% of the total manufacturing costs for sodium butyrate, which range from $1140 to $1940 per MT, depending on the scale (Fig. 6b). Figure 7 shows the breakdowns of manufacturing costs, which include raw materials (carbon source, corn steep liquor as the nitrogen source, NaOH, sulfuric acid, and solvent used in extraction), utilities (steam, water, electricity, and nitrogen gas), equipment depreciation and maintenance, and labor. The raw materials account for ~ 60% of the total manufacturing costs when dextrose (95% glucose, $450 per MT) is used, and decrease to 46% for corn (80% starch, $170 per MT) and 37% for corn husk (50% reducing sugars, $20 per MT). It is noted that the solvent (10% alamine 336 in 2-octanol) used in extraction has a high selectivity on butyric acid (with the distribution coefficient > 10 at ~ pH 5.5) and is immiscible in water (solubility < 1 ppm) [37], and thus the solvent replacement cost is relatively low in the extractive fermentation process. Utilities cost accounts for 10–12% while labor cost accounts for 9–11%. The facilities and equipment associated costs (maintenance and depreciation) account for ~ 38% of the total manufacturing costs for the corn husk plant, and decrease to ~ 33% for corn and ~ 22% for dextrose. The major equipment for the manufacturing process includes corn husk grinder, reactor for acid hydrolysis, activated-carbon adsorption column, fermenters (seed and FBB), liquid–liquid extraction column, and storage tanks, and with building and construction the total capital investment is ~ $6.4 MM for a 1000 MT plant.

The estimated manufacturing costs for 30% (w/v) sodium butyrate solution are ~ $1635/MT butyrate at 1000 MT/y and $1142/MT at 5000 MT/y, which are much lower than the current market price for the petroleum-derived butyrate (~ 1800/MT) and the manufacturing costs for using dextrose or corn as the substrate in fermentation. It is noted that the fermenter sizing in the analysis is based on a butyric acid productivity of 1 g L^−1^ h^−1^, which is a relatively conservative number as extractive fermentation usually can have at least two to threefold higher productivity compared to fermentation without in situ removal of inhibiting fermentation product, butyric acid [37]. Doubling the productivity to 2 g L^−1^ h^−1^ would reduce the fermentor size from ~ 120 to ~ 65 m^3^ for the 1000 MT plant, resulting in a 33% saving in the purchased fermenter, which can significantly reduce the total capital investment and return of investment. Nevertheless, the projected performance of the extractive fermentation process based on the earlier study with glucose as the substrate will need to be verified with corn husk hydrolysate before further development for commercial application.

## Discussion

Glucose and xylose are the two most abundant sugars in plant biomass. While glucose is widely used as the carbon source in industrial fermentations, its presence can inhibit the catabolism of other sugars such as xylose in fermentation. In this study, we first studied butyric acid production from glucose, xylose, and glucose/xylose mixture as carbon source, respectively, in fed-batch fermentations. In general, butyric acid production was higher from glucose than from xylose, which is consistent with the results reported before [14, 20, 39]. Glucose is usually catabolized via the Embden–Meyerhof–Parnas (EMP) pathway, while xylose is catabolized via the hexose monophosphate (HMP) pathway to pyruvate. Because of the requirement of extra energy in xylose transport, the net ATP yield from xylose was lower compared to that from glucose [13, 14]. Although glucose metabolism can provide more ATP for cell growth and butyric acid production, glucose-mediated carbon catabolite repression (CCR) could inhibit the consumption of non-glucose sugars such as xylose also present in the lignocellulosic biomass hydrolysate [12, 20, 40]. Xylose utilization by *C. tyrobutyricum* was inhibited by glucose in free-cell fermentation, although the CCR could be relieved by engineering the cell to overexpress three genes in the xylose catabolism pathway [12, 20, 40]. However, our results indicated that glucose mediated CCR would have little effect on butyric acid production from corn husk hydrolysate after cell adaptation in the FBB. Simultaneous utilization of glucose and xylose (1:1) present in a synthetic medium has also been reported for *C. tyrobutyricum* immobilized in a FBB, which presents another advantage, in addition to the significantly increased productivity and final product titer, over conventional free-cell fermentation [23].

Corn as one of the major agricultural sources in terms of quantity of biomass available has been widely used in industrial fermentation [41]. It was reported that a high butyric acid productivity of 6.78 g L^−1^ h^−1^ was obtained in a FBB system with corn meal containing ~ 75% corn starch, 20% corn fibers, and 1.5% protein as the substrate [17]. However, corn and other traditional substrates such as glucose and sucrose are expensive to use for butyric acid production. The high substrate cost is promoting the usage of economically available biomass feedstocks, including agricultural residues, food processing wastes, and energy crops [3, 24]. For example, corn fiber, an abundant by-product from corn processing, is also a promising fermentation feedstock. Butyric acid fermentation with corn fiber hydrolysate supplemented with corn steep liquor gave a high butyric acid yield of 0.47 g g^−1^ and a reactor productivity of 2.91 g L^−1^ h^−1^ [23]. Other biomass feedstocks including brown algae [19], wheat straw [22], sugarcane bagasse [25], and sorghum stalk [42] as low-cost substrates have also been studied for butyric acid production. Table 3 summarizes butyric acid production from various biomass hydrolysates in fermentation by *C. tyrobutyricum*. In general, a higher butyrate titer and productivity could be obtained in fermentations with a higher cell density and after adaptation in the bioreactor, especially with cells immobilized in a fibrous bed bioreactor [16–18]. The productivity obtained in this study could have been much higher if the FBB were allowed to continue to operate for additional batches to increase the cell density and further adapt cells in the reactor to better tolerate butyric acid. In extractive fermentation the butyric acid concentration in the fermentation broth would be maintained at ~ 10 g L^−1^ [37], which is much lower than the > 20 g L^−1^ of butyric acid produced in batch fermentation of corn husk hydrolysate. It is thus reasonable to believe that a butyric acid productivity of 1 g L^−1^ h^−1^ can be obtained in the extractive fermentation with corn husk hydrolysate.

**Table 3:** Comparison of butyric acid production from different substrates by *C*. *tyrobutyricum*

Substrate	Hydrolysis method	Fermentation mode	Titer (g L^−1^)	Yield (g g^−1^)	Productivity (g L^−1^ h^−1^)	References
Lignocellulosic biomass	0.04 M HCl/0.01–0.02 M H_2_SO_4_, 121 °C, 30 min, cellulases, 50 °C, 72 h	Batch in serum bottles	42.6	0.36	0.56	[20]
Sorghum stalk and beet molasses	50 mM citrate buffer, cellulases, 50 °C, 18 h	Batch in bioreactors	58.8	0.52	1.9	[42]
Wheat straw	1.4% H_2_SO_4_, 163 °C, 15 min, cellulases, 120 h	Batch in serum vials	20.0	0.33	0.21	[22]
SCB	0.1 M HCl, 121 °C, 10–15 min, cellulases, 50 °C, 24 h	Fed-batch in FBB	20.9	0.48	0.5	[25]
JA	0.01 M H_2_SO_4_, 121 °C, 30 min	Fed-batch in FBB	60.4	0.38	1.1	[18]
		Repeated-batch in FBB	27.5	0.44	2.75	
Brown algae	1.5% H_2_SO_4_, 121 °C, 1 h	Fed-batch in FBB	11.0	0.11	0.9	[19]
Cane molasses	5 M H_2_SO_4_, 60 °C, 2 h	Fed-batch in FBB	55.2	0.46	3.2	[16]
Corn meal	0.6% amylase, 60 °C	Fed-batch in FBB	46.0	0.45	6.8	[17]
Corn fibre	0.25 M HCl, 121 °C, 45 min	Fed-batch in FBB	29.0	0.47	2.9	[23]
Corn husk	0.4 M H_2_SO_4_, 110 °C, 6 h	Repeated batch in FBB	20.8	0.39	0.42	This study

*FBB* fibrous bed bioreactor, *JA* Jerusalem artichoke, *SCB* sugarcane bagasse

Corn husk is a rarely studied agricultural residue from the corn field. It contained (w/w) 40% cellulose, 45% hemicellulose, 7% lignin, 2% protein, and 3% ash [43]. As demonstrated in the present study, corn husk can be readily hydrolyzed with dilute sulfuric acid at 110 °C to reducing sugars at a high yield of ~ 50% (w/w) without requiring enzymatic treatments, and is more economical to use as substrate for butyrate production than traditional fermentation substrates such as glucose and corn starch. Corn husk also has a cost advantage over other more extensively studied lignocellulosic biomass including sugarcane bagasse and corn fiber, which after expensive pretreatments and enzymatic hydrolysis would cost more than corn and dextrose as the fermentation substrate. After acid hydrolysis and detoxification, the corn husk hydrolysate contained ~ 45 g L^−1^ total reducing sugars, 0.5 g L^−1^ HMF, and trace amounts of furfural and other degradation compounds, which showed little or no inhibition effects on cell growth and butyric acid production. It is known that phenolics compounds generated from lignin degradation during acid pretreatment of some lignocellulosic biomass were highly toxic to cells and strongly inhibited the fermentation [20, 44]. However, corn husk has a relatively low lignin content and its acid hydrolysate was thus not toxic after detoxification with activated charcoal. Therefore, corn husk hydrolysate, after detoxification, was a good substrate for butyric acid fermentation by *C. tyrobutyricum* in the FBB without any notable glucose induced CCR. In contrast, the consumption of xylose in corn fiber and sugarcane bagasse hydrolysates was significantly slower than glucose and was inhibited by inhibitors present in the hydrolysate [23, 25].

The cost to produce encapsulated sodium butyrate from petroleum-derived butyrate ($1800 per MT) for animal feed application is about US $480 per MT of the product containing 30% sodium butyrate, which is currently sold at US $2380–$3174 per MT. A similar product can be produced from corn husk at US $823–$971 per MT, assuming a similar encapsulation process cost for the fermentation-derived sodium butyrate. With the large gross margin of more than $1400 per MT, the process is highly profitable and economically attractive.

## Conclusion

Hydrolysis of 10% (w/v) corn husk in 0.4 M H_2_SO_4_ at 110 °C for 6 h yielded 45.06 g L^−1^ total reducing sugars, which could be converted to butyric acid by *Clostridium tyrobutyricum* immobilized in a FBB. Stable production of butyric acid of ~ 21 g L^−1^ with an average yield of 0.39 g g^−1^ was achieved in the repeated-batch fermentation, demonstrating that corn husk hydrolysate can be used as an economical substrate for butyric acid fermentation by *C. tyrobutyricum.* Sodium butyrate at a high concentration of > 30% (w/v) can be produced economically in an extractive fermentation process and used to manufacture encapsulated sodium butyrate for animal feed application.

## Methods

### Corn husk

Corn husk, obtained from the farmland of Xiaogan (Hubei, China) was dried at 60 °C to less than 5% moisture and ground into fine powder (particle size: 50–100 μm) by the micro-milling (DJ-04 Model, Dianjiu Traditional Chinese Medicine Machinery Manufacturing Co. Ltd., Shanghai, China). Corn husk powder was further dried at 60 °C to a constant weight and stored until use.

### Acid hydrolysis of corn husk

The hydrolysis of corn husk powder was carried out in 250-mL glass bottles with sulfuric acid. The orthogonal experimental design was used to evaluate process parameters, including sulfuric acid concentration (0.2, 0.3, 0.4 or 0.5 M), corn husk to sulfuric acid ratio (w:v) (1:10, 1:15, 1:20 or 1:25), temperature (90, 100, 110 or 120 °C), and processing time (2, 4, 6 or 8 h) for achieving the highest production of total reducing sugars in corn husk hydrolysate. The orthogonal experiment design can dramatically reduce the total number of experiments required to obtain the optimal process conditions [45, 46], from 256 possible combinations (4 × 4 × 4 × 4) to 16 for the 4 factors at 4 levels studies. After acid treatment, hydrolysates were clarified by centrifuging at the 1000 rpm for 5 min and supernatant samples were stored in 2-mL cryogenic storage vials for analysis of the total reducing sugars. The optimal conditions of acid hydrolysis were selected according to the yield of total reducing sugars.

### Detoxification of corn husk hydrolysate

Corn husk hydrolysate was mixed with activated carbon (1%, w/v) with mechanical agitation (800 rpm) at 30 °C for 30 min to remove toxic chemicals, such as pigments, HMF and phenolic compounds that might inhibit cell growth and butyric acid fermentation. Samples were taken after 30-min treatment to assay the total reducing sugars and HMF concentrations.

### Bacterial stain, media and cultivation

*Clostridium tyrobutyricum* ATCC25755 was cultured at 37 °C in 100-mL serum bottles (50-mL working volume) containing the reinforced clostridial growth medium (CGM), which contained (g/L): 20 glucose, 2 yeast extract, 4 peptone, 2 (NH_4_)_2_SO_4_, 1 K_2_HPO_4_, 1 KH_2_PO_4_, 0.1 MgSO_4_·7H_2_O, 0.015 FeSO_4_·7H_2_O, 0.015 CaCl_2_·2H_2_O, 0.01 MnSO_4_·H_2_O, 0.02 CoCl_2_·6H_2_O, 0.002 ZnSO_4_·7H_2_O [39]. All media were purged with N_2_ for 30 min and autoclaved at 121 °C for 30 min.

### Free-cell fermentations

Free-cell fermentations were conducted using glucose and xylose as carbon sources in 5-L fermentors (2-L working volume) at 37 °C, agitated at 150 rpm and pH was controlled at 6.0 with 30% NH_3_·H_2_O. All media were purged with N_2_ for 30 min and sterilized at 121 °C for 30 min. Cell growth and product formation throughout the fermentation were monitored with samples taken every 4 h.

### Fermentations in fibrous-bed bioreactor

Fermentations were also carried out with cells immobilized in a fibrous-bed bioreactor (FBB) following the procedures described previously [13]. The FBB was made of a glass column packed with a spirally wound cotton towel (18 × 30 cm, ~ 0.5 cm in thickness, 95% porosity) overlaid with a stainless steel mesh. The FBB was connected to a 5-L stirred-tank fermentor with a recirculation pump, and the bioreactor system was operated with a total liquid volume of 2 L at 37 °C, agitated at 150 rpm, and pH controlled at 6.0 with 30% NH_3_·H_2_O via an auto-sensing and dosing system. Before use, the bioreactor containing the medium was sterilized by autoclaving at 121 °C for 30 min, and then flushed with N_2_ for about 30 min. To start the fermentation, the fermentor was inoculated with 100 mL cells cultured in serum bottles and allowed to grow to reach a cell density of ~ 4.0 g L^−1^. The medium was then recirculated between the fermentor and the FBB at a low flow rate for cell immobilization in the fibrous bed. After ~ 2 days when most of the cells had been immobilized, the fermentation broth in the fermentor was removed and replaced with fresh medium for fermentation kinetics studies. The fermentation was first studied with glucose, xylose, and the mixture of glucose and xylose (1:1, w/w), respectively, as carbon sources in the fed-batch mode by pulse feeding a concentrated sugar solution when sugars were almost depleted in the fermentation broth. The pulse feeding was repeated until the fermentation stopped producing butyric acid because of product inhibition. Repeated batch fermentations with corn husk hydrolysate were then studied to evaluate the kinetics and possible effects of hydrolysate inhibitors on long-term process performance. Samples were taken every 4 h for analyses of cell density, glucose, xylose, butyric acid, and acetic acid.

### Analytical methods

The total reducing sugars after acid hydrolysis was measured by the dinitrosalicylic (DNS) colorimetric method [47, 48]. Briefly, 0.5 mL DNS mixture solvent and 1 mL corn husk hydrolysate sample were heated at 100 °C for 15 min, and cooled down and diluted into 10 mL. The value of the mixture was measured at 540 nm (OD_540_) in a spectrophotometer. Glucose in corn husk hydrolysate after acid pretreatment was evaluated by using a SBA Biosensor Analyzer (Biology Institute of Shandong Academy of Science. Shandong, China). HMF in the hydrolysate was analyzed at 272 nm (OD_272_) with a spectrophotometer. Glucose, xylose, butyric acid, and acetic acid in the fermentation broth were analyzed with a high-performance liquid chromatography (HPLC) system (Agilent Technology, USA) following the method described previously [12, 49]. Cells in broth samples were centrifuged, washed twice, and resuspended before the optical density at 600 nm (OD_600_) was measured with a spectrophotometer.

## Abbreviations

FBB: fibrous bed bioreactor
HPLC: high-performance liquid chromatography
HMF: 5-hydroxymethylfurfural
EMP: Embden–Meyerhof–Parnas
HMP: hexose monophosphate
CCR: carbon catabolite repression
CGM: clostridial growth medium
DNS: dinitrosalicylic
SCB: sugarcane bagasse
JA: Jerusalem artichoke
CHH: corn husk hydrolysate
